# Potentially inappropriate medications at discharge: Prevalence, predictors, and their association with early readmission and emergency department visits in older adults

**DOI:** 10.1371/journal.pone.0329778

**Published:** 2025-08-12

**Authors:** Watcharasak Sombut, Kanthida Methaset, Arom Jedsadayanmata

**Affiliations:** 1 Sirindhorn College of Public Health Chonburi, Faculty of Public Health and Allied Health Sciences, Praboromarajchanok Institute, Muang, Chonburi, Thailand; 2 Department of Pharmacy Services, Thammasat University Hospital, Khong Luang, Pathum Thani, Thailand; 3 Faculty of Pharmacy, Thammasat University, Khong Luang, Pathum Thani, Thailand; Children's National Hospital, George Washington University, UNITED STATES OF AMERICA

## Abstract

**Background:**

Evidence on the association between potentially inappropriate medications (PIMs) and adverse outcomes after hospital discharge remains limited and contradictory. This study aimed to determine the prevalence, predictors, and impact of PIMs at discharge on early unplanned readmissions and emergency department (ED) visits in older adults.

**Methods:**

This retrospective cohort study analyzed electronic medical records of older patients discharged from a tertiary-care hospital to home. Patients were followed for 90 days to assess unplanned readmissions and ED visits. Multiple Cox regression and parametric survival analysis determined the association between PIMs and early readmissions/ED visits.

**Results:**

Among 4,012 older patients, 2,299 (57.3%) were discharged with at least one PIM. Factors independently associated with PIM use included a higher Charlson Comorbidity Index (OR 1.08, 95% CI 1.01–1.15, p = 0.02), longer hospital stay (OR 1.01, 95% CI 1.00–1.02, p = 0.01), and a greater number of discharge medications (OR 1.26, 95% CI 1.24–1.29, p < 0.001). Within 90 days post-discharge, unplanned readmissions or ED visits occurred in 183 of 2,299 patients (7.96%) with PIMs and 89 of 1,713 (5.20%) without PIMs. In multivariable Cox regression, PIM use was associated with a non-significant increase in the risk of unplanned readmission/ED visits (HR 1.15, 95% CI 0.87–1.51, p = 0.32), a finding consistent across parametric survival models using Weibull, exponential, lognormal, and log-logistic distributions.

**Conclusion:**

PIMs were highly prevalent in older patients at discharge, with comorbidity burden, the duration of hospital stay, and polypharmacy as significant predictors. However, PIMs at discharge were not significantly associated with early unplanned readmissions or ED visits.

## Introduction

The proportion of older populations is increasing in many countries worldwide [1]. Older individuals often face issues related to medication use due to a combination of chronic illnesses such as diabetes mellitus, heart diseases, and chronic kidney disease, as well as age-related physiological changes, weakened immune systems, and increased exposure to medications. These factors increase the risk of adverse drug events and complex treatment needs [2–4]. Studies indicate that adverse drug events, non-compliance with medication regimens, and medication errors lead to hospital admissions and readmissions resulting from medication-related issues in older adults [5–8]. Adverse drug events in older adults frequently result from the use of inappropriate medications, also known as potentially inappropriate medications (PIMs) [9]. According to the Beers criteria for PIMs, the use of first-generation antihistamines or antidepressants with high anticholinergic properties increases the risk of anticholinergic side effects, contributing to a higher risk of falls, delirium, and dementia [9]. Similarly, the use of benzodiazepines raises the risk of cognitive impairment, delirium, falls, and fractures, while non-COX-2-selective NSAIDs increase the risk of gastrointestinal ulcers or bleeding in older adults [9]. Systematic reviews and meta-analyses have found that the use of PIMs in older patients increases the risk of adverse drug events, emergency department visits, and hospital admissions [10–12], compared to patients who do not receive PIMs. However, most studies of PIMs were conducted in hospitalized, community-dwelling, and nursing home older patients.

A literature review reveals that studies tracking health outcomes following the use of PIMs during transitional care are limited and often contradictory. Some studies have shown that PIMs at discharge are associated with an increased risk of unplanned readmissions or outpatient visits [13–16]. However, several studies have found no such association [17–20], and a few have even reported a reduced risk of readmission post-discharge with PIMs [21,22]. These discrepancies may stem from various factors and limitations specific to each study, such as sample size, eligibility criteria, PIM assessment methods, follow-up duration, and outcome measurements.

This study aimed to determine the prevalence and predictive factors of PIMs at discharge among older adults. It also investigated the association between PIMs at discharge and unplanned readmissions or emergency department (ED) visits within 90 days post-discharge, using both semiparametric and parametric survival models. The findings may help optimize prescribing practices and improve the quality of care transitions for older individuals.

## Methods

### Study setting and population

This retrospective cohort study was conducted at an 800-bed tertiary-care teaching hospital in central Thailand. The hospital trains medical students, residents, and fellows while delivering comprehensive healthcare services. It houses specialized departments, including cardiology, neurology, oncology, and various surgical specialties. The facility also operates multiple intensive care units (ICUs), such as medical, surgical, and cardiovascular ICUs. In addition to inpatient care, the hospital offers specialized outpatient clinics that support chronic disease management and specialty consultations. These clinics ensure continuity of care by providing follow-up services for patients requiring long-term treatment. The hospital’s outpatient pharmacy also serves as the primary source for prescription refills.

Participants were identified through electronic health records (EHRs) of patients hospitalized for at least 24 hours and later discharged home from medical services. The recruitment period extended from September 2021 to September 2023. Eligible patients were those aged 60 years or older at the time of discharge. For individuals with multiple hospital admissions during the recruitment period, only the first admission was considered as their index hospitalization.

### Variables and data collection

Data were collected from EHRs of inpatient and outpatient departments and pharmacy services, using a unique identification code to link data for each patient and admission. Key variables included patient demographics, discharge services, principal diagnosis, comorbidities, and medication orders. The dataset was provided by the Information Technology Department of the study hospital on July 17, 2024, and was allowed for use only in the present research project. The authors did not have access to information that could identify individual participants during or after data collection.

**Fig 1:**
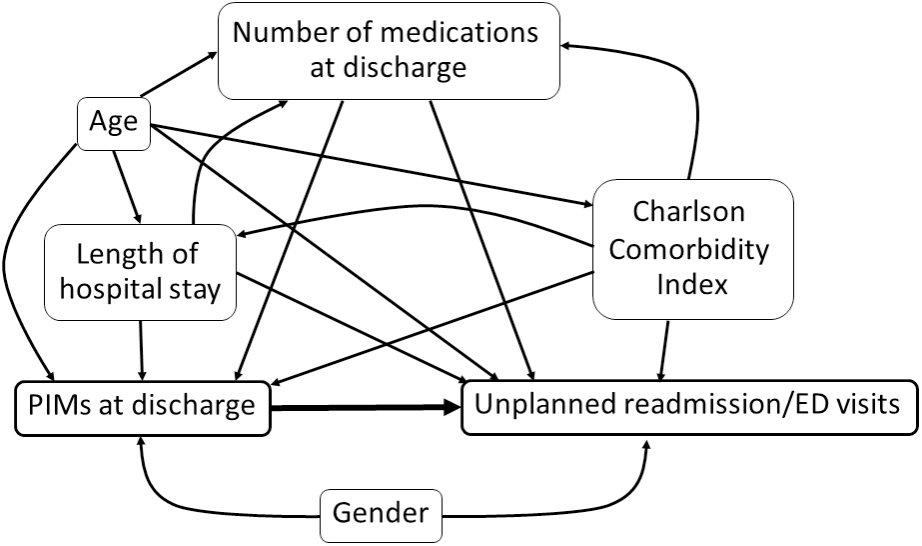
Causal directed acyclic graph illustrating hypothetical association among covariates, PIMs, and unplanned early readmission/ED visits.

Fig 1 shows the causal directed acyclic graph illustrating the hypothetical association among covariates in the study, PIMs, and unplanned early readmission/ED visits. PIMs at discharge among participants were identified based on the 2023 American Geriatrics Society (AGS) Beers Criteria [9]. The identification process followed two main steps. First, medications classified as “drugs to avoid in most older patients” with a strong recommendation (listed in Table 2 of the 2023 criteria) were considered PIMs. The presence of any of these medications in the study population was categorized as a PIM. Second, the medications classified as “drugs to avoid in older adults with specific diseases or syndromes” due to drug-disease or drug-syndrome interactions that may exacerbate the condition (listed in Table 3 of the 2023 criteria) were assessed. For each patient prescribed any of these medications, drug-disease or drug-syndrome interactions were evaluated based on their principal diagnosis and comorbidities recorded in the EHRs. The present study did not include medications listed in Table 4 of the 2023 criteria, which require clinical judgment for appropriate use, as the criteria emphasize caution rather than absolute avoidance in determining PIM status.

**Table 1:** Baseline characteristics of study participants based on the presence of PIMs at discharge (N = 4012).

Characteristics	With PIMs (N = 2,299)	Without PIMs (N = 1,713)	P-value*
Age, mean (SD)	74.98 (9.24)	73.75 (9.04)	<0.0001
Male, n (%)	1,076 (46.80)	843 (49.21)	0.13
Married, n (%)	1,522 (66.20)	1,139 (66.49)	0.85
CCI score			
Mean (SD)	0.84 (1.18)	0.69 (1.11)	< 0.0001
CCI score = 0, n (%)	1,146 (49.85)	1,024 (59.79)	<0.001
CCI score = 1, n (%)	634 (27.58)	355 (20.72)	
CCI score = 2, n (%)	401 (17.44)	263 (15.35)	
CCI score = 3, n (%)	75 (3.26)	49 (2.86)	
CCI score ≥ 4, n (%)	43 (1.87)	22 (1.28)	
Number of medications at discharge			
Median (IQR)	9 (6-12)	5 (3-8)	<0.0001
Mean (SD)	9.01 (4.03)	5.49 (3.61)	<0.0001
Length of hospital stay (days)			
Median (IQR)	6 (3-13)	4 (2-8)	<0.0001
Mean (SD)	10.41 (13.74)	6.62 (8.33)	<0.0001

* The two groups were compared using an independent t-test for means, a Wilcoxon rank-sum test for medians, and a Chi-square test for proportions.

*Abbreviations:* CCI, Charlson Comorbidity Index; PIMs, potentially inappropriate medications.

**Table 2:** Prevalence of PIMs at discharge by (A) the number of PIMs per patient and (B) the top 10 most prevalent PIMS (N = 4012).

(A) Number of PIMs per patient prescribed at discharge	
Number of PIMs per patient	Prevalence, n (%)
0 PIM	1,713 (42.70)
1 PIM	1,468 (36.58)
2 PIMs	621 (15.48)
3 PIMs	182 (4.54)
≥ 4 PIMs	28 (0.70)
**(B) The top 10 most prevalent PIMs at discharge**	
**Medications**	**Prevalence, n (%)**
1. Proton pump inhibitors	1,020 (25.42)
2. Aspirin	672 (16.75)
3. Lorazepam	443 (11.04)
4. Warfarin	202 (5.03)
5. Quetiapine	191 (4.76)
6. Doxazosin	180 (4.49)
7. Glipizide	135 (3.36)
8. Clonazepam	69 (1.72)
9. Metoclopramide	50 (1.25)
10. Alprazolam	42 (1.05)
10. Orphenadrine	42 (1.05)

*Abbreviation:* PIMs, potentially inappropriate medications.

**Table 3:** Factors associated with PIMs prescribed at discharge (N = 4012).

Variables*	Unadjusted OR (95% CI)	P-value	Adjusted OR** (95% CI)	P-value
Age	1.01 (1.01-1.02)	< 0.01	1.00 (0.99-1.01)	0.96
Male	0.91 (0.80-1.03)	0.13	0.93 (0.81-1.06)	0.27
CCI score	1.13 (1.07-1.20)	< 0.01	1.08 (1.01-1.15)	0.02
LOS	1.04 (1.03-1.05)	< 0.01	1.01 (1.00-1.02)	0.01
Number of medications at discharge	1.27 (1.25-1.30)	< 0.01	1.26 (1.24-1.29)	< 0.01

* Male were entered in regression as a categorical variable with male coded as 1 and female coded as 0. All other variables were entered as continuous numeric variables, with effect sizes interpreted as ORs per one-unit increase in each variable.

** The area under the receiver operating characteristic (ROC) curve for the multivariable model is 0.7508.

*Abbreviations*: CCI, Charlson Comorbidity Index; LOS, length of hospital stay; PIMs, potentially inappropriate medications.

**Table 4:** Association of PIMs with unplanned readmission/ED visits in Cox proportional hazard regression (N = 4012).

Variables*	Univariable analysis		Multivariable analysis	
	Crude HR (95% CI)	P-value	Adjusted HR (95% CI)	P-value
Age	1.03 (1.02-1.05)	< 0.01	1.03 (1.01-1.04)	< 0.01
Male	1.12 (0.89-1.43)	0.34	1.17 (0.92-1.49)	0.19
CCI	1.06 (0.96-1.16)	0.25	1.04 (0.95-1.15)	0.40
LOS	1.01 (1.01-1.02)	< 0.01	1.01 (1.00-1.01)	0.02
Number of medications at discharge	1.09 (1.06-1.12)	< 0.01	1.07 (1.03-1.10)	< 0.01
PIMs at discharge	1.55 (1.21-2.00)	< 0.01	1.15 (0.87-1.51)	0.32

* Male and PIMs at discharge variables were entered in regression as categorical variables with the attributes in the Table coded as 1 and the reference category coded as 0. All other variables were entered as continuous numeric variables, with effect sizes interpreted as hazard ratios per one-unit increase in each variable.

*Abbreviations:* CCI, Charlson Comorbidity Index; ED, emergency department; LOS, length of hospital stay; PIMs, potentially inappropriate medications

Early readmission and ED visits were identified from the EHRs when participants were recorded to visit the ED within 90 days after discharge. Participants who were hospitalized after the ED visits were classified as unplanned readmissions. Those participants who were not readmitted or visited the ED within 90 days were censored in the survival analysis.

The Charlson Comorbidity Index (CCI) was used to quantify patients’ comorbidity burden. It was derived from ICD-10 diagnosis codes recorded at hospital discharge in the EHRs. This study used the *charlson* command in Stata, which follows the algorithm developed by Quan and colleagues for mapping diagnoses to 17 comorbid conditions [23]. Each condition carries a specific weight based on its mortality risk, and the total score was calculated as the sum of these weights. The CCI score was then entered as a numeric variable and modeled as continuous in regression analyses.

### Statistical analysis

The prevalence of each PIM was determined by calculating the proportion of participants prescribed the medication relative to the total study population, expressed as a percentage. The ten most frequently prescribed PIMs were ranked by prevalence.

To identify factors associated with PIM prescriptions at discharge, logistic regression analysis was conducted, with results presented as odds ratios (ORs) and 95% confidence intervals (CIs). All variables, except gender, were included as continuous predictors—specifically age, CCI score, LOS, and the number of medications at discharge. The predictive performance of the multivariable model was assessed using the area under the receiver operating characteristic (ROC) curve.

The association between PIM use and unplanned early readmission or ED visits was examined using Cox proportional hazards regression. Unplanned early readmission was defined as hospitalizations occurring within 90 days post-discharge following an ED visit. Participants without unplanned readmission or ED visits were censored at 90 days. Hazard ratios (HRs) with 95% CIs were reported. The proportional hazards assumption was assessed using Schoenfeld residuals, with a p-value >0.1 indicating that the assumption was met.

To explore model robustness, parametric survival analyses were conducted using Weibull, exponential, lognormal, and log-logistic distributions. Associations were reported as model coefficients and the model fit was assessed with the Akaike Information Criterion (AIC) and Bayesian Information Criterion (BIC).

Multicollinearity among covariates in multivariable models was assessed using the variance inflation factor (VIF), with variables exceeding a VIF threshold of 5 excluded from analysis.

All analyses were performed using Stata 18 (StataCorp, College Station, TX). Statistical significance was set at p < 0.05, and all hypothesis tests were two-sided.

### Ethical considerations

The Human Research Ethics Committee of Thammasat University (Science) exempted this study from requiring ethics approval (Document number: COE 002/2567, Research Project Code: 66PH160). Given the retrospective study design and minimal risk to participants, the ethics committee waived the requirement for informed consent. All data were fully anonymized, and data collection, access, and handling were conducted in accordance with the Declaration of Helsinki, institutional regulations, and applicable ethical guidelines to ensure patient confidentiality.

## Results

### Baseline characteristics of the study participants

**Fig 2:**
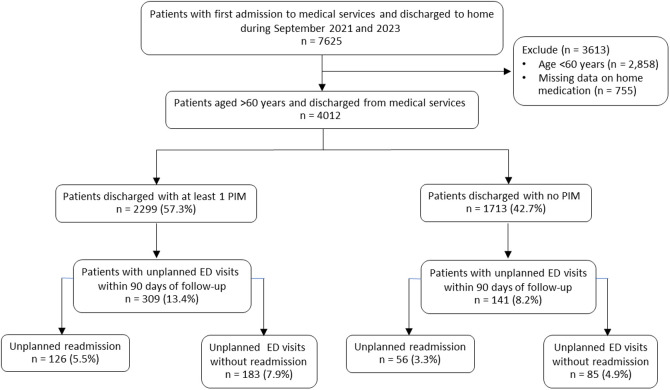
Participant flow chart. ED, emergency department; PIM, potentially inappropriate medication.

The participant flow chart is presented in Fig 2. The study population comprised 4,012 older adults aged ≥60 years, with 2,299 (57.3%) receiving at least one PIM and 1,713 (42.7%) discharged without PIMs.

Table 1 shows the baseline characteristics of study participants stratified by the presence of PIMs at discharge. Participants receiving PIMs were older than those without PIMs (mean age: 74.98 ± 9.24 vs. 73.75 ± 9.04 years, p < 0.0001). There was no significant difference in gender distribution, with males accounting for 46.8% of the PIM group and 49.2% of the non-PIM group (p = 0.13). Comorbidity burden, assessed using the CCI, was higher in the PIM group (mean CCI score: 0.84 ± 1.18 vs. 0.69 ± 1.11, p < 0.0001), indicating a greater prevalence of chronic conditions. There was significant heterogeneity in the distribution of CCI scores between the two groups (p < 0.001).

Polypharmacy was more prevalent among participants receiving PIMs. The median number of medications at discharge was significantly higher in the PIM group (9 [IQR: 6–12] vs. 5 [IQR: 3–6], p < 0.0001). Additionally, the mean number of medications at discharge was higher in the PIM group (9.01 ± 4.03 vs. 5.49 ± 3.61, p < 0.0001).

The LOS was also significantly longer among patients with PIMs. The median LOS was 6 days (IQR: 3–13) for the PIM group compared to 4 days (IQR: 2–8) for the non-PIM group (p < 0.0001). The mean LOS followed a similar trend (10.41 ± 13.74 vs. 6.62 ± 8.33 days, p < 0.0001).

### Prevalence of PIMs at hospital discharge

Table 2 highlights the prevalence of PIMs prescribed to older patients at discharge according to the AGS 2023 updated Beers Criteria^®^. Among the 4,012 patients in the study, 2,299 (57.3%) were discharged with at least one PIM. The majority of these patients (36.58%) had a single PIM prescribed, while 15.48% had two PIMs, 4.54% had three PIMs, and a smaller proportion (0.70%) had more than three PIMs at discharge. The most frequently prescribed PIMs were proton pump inhibitors (PPIs) (25.42%), followed by aspirin (16.75%), lorazepam (11.04%), and warfarin (5.03%).

### Factors associated with PIMs at discharge

Factors associated with the prescription of PIMs are shown in Table 3. In the unadjusted analysis, older age (OR 1.01, 95% CI 1.01–1.02, P < 0.01), higher CCI score (OR 1.13, 95% CI 1.07–1.20, P < 0.01), longer LOS (OR 1.04, 95% CI 1.03–1.05, P < 0.01), and a higher number of medications at discharge (OR 1.27, 95% CI 1.25–1.30, P < 0.01) were significantly associated with an increased likelihood of PIM prescriptions. After adjusting for other covariates, the CCI score remained significantly associated with PIMs, with each unit increase in the CCI score increasing the odds of receiving a PIM by 8% (adjusted OR 1.08, 95% CI 1.01–1.15, P = 0.02). Similarly, an additional day in LOS increased the odds of PIMs prescription at discharge by 4% (adjusted OR 1.04, 95% CI 1.03–1.07, P < 0.01). The strongest predictor remained the number of medications at discharge, with each additional medication increasing the likelihood of receiving a PIM by 26% (adjusted OR 1.26, 95% CI 1.24–1.29, P < 0.01).

### Association of PIMs at discharge and unplanned early readmission/ED visits

Table 4 presents the association of PIMs at discharge with unplanned readmission or ED visits using Cox regression analysis. In univariable analysis, PIMs were associated with increased hazard for unplanned readmission/ED visits (HR 1.55, 95%CI 1.21 to 2.00, p < 0.01). However, in multivariable analysis, PIM use was not a significant predictor of unplanned readmission/ED visits after adjusting for other variables (adjusted HR 1.15, 95%CI 0.87 to 1.51, p = 0.32).

In parametric survival models (Table 5), across the Weibull, exponential, lognormal, and log-logistic models, the number of medications at discharge, age, and LOS were consistently associated with unplanned readmission/ED visits. However, PIMs at discharge were not significantly associated with the outcome in parametric models. Model fit statistics (AIC and BIC) varied across the models, with the log-logistic model showing the lowest AIC and BIC values.

**Table 5:** Association of PIMs at discharge with unplanned readmission/ED visits in parametric survival models (N = 4012).

	Weibull		Exponential		Lognormal		Log-logistic	
Variables*	Coefficients	p-value	Coefficients	p-value	Coefficients	P-value	Coefficients	P-value
Age	0.027	<0.01	0.027	<0.01	−0.046	<0.01	−0.043	<0.01
Male	0.161	0.18	0.163	0.18	−0.254	0.22	−0.265	0.18
CCI score	0.041	0.28	0.042	0.28	−0.077	0.32	−0.068	0.30
LOS	0.007	<0.01	0.007	<0.01	−0.021	<0.01	−0.014	<0.01
Number of medications at discharge	0.064	<0.01	0.064	<0.01	−0.106	<0.01	−0.104	<0.01
PIMs at discharge	0.140	0.31	0.141	0.31	−0.205	0.37	−0.222	0.31
AIC	2747.898		2816.542		2725.419		2744.455	
BIC	2798.274		2860.622		2775.796		2794.832	

* Male and PIMs at discharge variables were entered in regression as categorical variables with the attributes in the Table coded as 1 and the reference category coded as 0. All other variables were entered as continuous numeric variables; coefficients are exponentiated and interpreted as hazard ratios in the Weibull and exponential models, and as time ratios in the log-normal and log-logistic models, per one-unit increase in each variable.

*Abbreviations:* AIC, Akaike Information Criteria; BIC, Bayesian Information Criteria; CCI, Charlson Comorbidity Index; ED, emergency department; LOS, length of hospital stay; PIMs, potentially inappropriate medications.

## Discussion

In the present study of older adults discharged from a tertiary care hospital, PIMs were highly prevalent, with more than half of the patients (57.3%) receiving at least one PIM at discharge. Despite concerns that PIMs may contribute to adverse outcomes, the study’s findings indicate that PIM use was not significantly associated with an increased risk of unplanned readmissions or ED visits within 90 days. This result remained consistent across Cox proportional hazards regression and parametric survival models, suggesting that while PIM prescribing is common, its direct impact on short-term healthcare utilization may be limited. Instead, factors such as comorbidity burden, length of hospital stay, and polypharmacy appear more influential in predicting early post-discharge outcomes.

Using the 2023 Beers Criteria, the present study identified a high prevalence of PIMs at discharge from medical services (57.3%), raising concerns about medication safety during care transitions. This finding is comparable to previous reports on PIM prevalence at hospital discharge [13,19,24,25]. The reported prevalence of PIMs at discharge varies, with studies showing 66% in Canada [23], 43.3% in Taiwan [13], 32.2% in Japan [19], and 87.2% in Portugal [25]. Differences in these estimates may be due to variations in study populations and how the Beers Criteria were applied. Nevertheless, the consistently high prevalence underscores the need for careful medication management at discharge to mitigate risks associated with inappropriate prescribing in older adults.

In the present study, older age, higher comorbidity burden, and polypharmacy emerged as key predictors of PIM prescriptions at discharge. Polypharmacy, defined as the use of five or more medications, is a well-established risk factor for PIM exposure and adverse clinical outcomes in older adults [13,17,20]. In the present study, each additional medication prescribed at discharge was associated with a 26% increase in the odds of receiving a PIM, even after adjusting for other covariates (Table 3), underscoring the importance of targeting polypharmacy as a modifiable risk factor. Furthermore, one prior study reported that PIMs at admission were significantly associated with PIMs prescribed at discharge, suggesting that early identification of PIMs during hospitalization may help reduce inappropriate prescribing at care transitions [26]. Given these risks, systematic medication reviews and deprescribing strategies should be integrated into routine hospital care. Interdisciplinary healthcareteams, including physicians, pharmacists, and nurses, will optimize medication regimens to minimize PIM use and protect this vulnerable population [27,28].

The present study found no significant association between PIM use at discharge and early readmission or ED visits. This finding remained consistent across semi-parametric and parametric survival models, suggesting that it is unlikely due to model misspecification. While this strengthens the validity of the current study results, it does not necessarily mean that PIMs have no impact on hospital returns. Several factors could explain the null association. The 90-day follow-up period may have been too short to capture long-term adverse effects such as cognitive decline, falls, or organ toxicity, which often develop gradually. Additionally, outpatient providers’ post-discharge medication reviews and deprescribing efforts may have mitigated potential harm before readmission. Moreover, hospital readmissions are multifactorial, often influenced by comorbidities, social determinants, and healthcare access, which may overshadow the specific effects of PIMs. Not all PIMs carry equal risk; grouping them may have diluted the impact of higher-risk medications, thus underestimating their effects. Future research should explore longer follow-up periods, larger sample sizes, and subgroup analyses focusing on high-risk PIMs to clarify their impact on hospital returns.

Although the present study did not establish a significant association between PIM use at discharge and short-term adverse outcomes, the importance of minimizing PIMs in geriatric care remains paramount. PIMs are well-documented risk factors for adverse drug reactions, drug-drug interactions, and medication-related hospitalizations, many of which may manifest beyond a 90-day follow-up period [13–16]. Reducing PIM use is crucial for preventing polypharmacy and prescribing cascades, both of which contribute to long-term morbidity in older adults. While a causal relationship with early readmission was not evident at the population level, certain high-risk subgroups—such as frail patients or those with multiple comorbidities—may still experience significant harm from inappropriate prescribing. Moreover, deprescribing efforts align with global medication safety initiatives, including those led by the World Health Organization [29] and the Choosing Wisely campaign [30]. Given these implications, optimizing medication regimens at discharge remains essential for improving patient safety and reducing the high burden of PIMs among older adults [31,32]. Future research should identify which specific types of PIMs or patient subgroups are most vulnerable, allowing for more targeted deprescribing strategies.

This study has several limitations. First, while the present research utilized EHRs, including principal diagnoses and comorbidities, to support the identification of potential indications for certain medications, the possibility of PIM misclassification remained. Some medications classified as PIMs—such as proton pump inhibitors or warfarin—may have been appropriately prescribed based on factors not fully captured in the dataset, such as treatment duration, dosing appropriateness, or specific patient-level risks (e.g., prior gastrointestinal bleeding, fall risk, frailty). Additionally, coding limitations might not reflect the clinical rationale for prescribing decisions. As a result, the study findings may overestimate the prevalence of inappropriate prescribing. Future studies with detailed clinical notes or chart reviews could improve the accuracy of PIM identification. Second, the observational nature of the present research posed a question of confounding by indication. This would typically bias results toward a false positive association between PIMs and adverse outcomes since they are often prescribed to high-risk patients already prone to readmission. The fact that this study found no significant association despite this potential bias suggests that if an effect exists, it is likely small. Last, the present study used data derived from a single tertiary-care hospital, so the generalizability of the findings to other settings and populations may need confirmation; nevertheless, the study findings aligned with previous reports [17–20].

## Conclusion

PIMs were found to be highly prevalent among older adults at hospital discharge, with comorbidity burden, length of hospital stay, and polypharmacy identified as significant predictors. Despite this high prevalence, the presence of PIMs at discharge was not independently associated with early unplanned readmissions or ED visits in adjusted semiparametric and parametric survival models. Future research should focus on examining specific classes of PIMs and their clinical consequences to inform targeted deprescribing strategies for high-risk older adults.
